# Macroscopic inhibition of DNA damage repair pathways by targeting AP-2α with LEI110 eradicates hepatocellular carcinoma

**DOI:** 10.1038/s42003-024-05939-7

**Published:** 2024-03-19

**Authors:** Chenchen Wang, Zhenjun Zhao, Yudong Zhao, Jie Zhao, Lei Xia, Qiang Xia

**Affiliations:** 1 Department of Liver Surgery, Renji Hospital, Shanghai Jiao Tong University School of Medicine, Shanghai, China; 2 Shanghai Engineering Research Center of Transplantation and Immunology, Shanghai, China; 3 Shanghai Institute of Transplantation, Shanghai, China

**Keywords:** Cancer genomics, Cancer

## Abstract

DNA damage repair (DDR) genes are known to be closely associated with the progression of Hepatocellular carcinoma (HCC). Here we report a unique cluster of “deletion-up” genes in HCC, which are accordantly overexpressed in HCC patients and predict the unfavorable prognosis. Binding motif analysis and further validation with ChIP-qPCR unveil that the AP-2α directly modulate the transcription of critical DNA repair genes including *TOP2A*, *NUDT1*, *POLD1*, and *PARP1*, which facilitates the sanitation of oxidized DNA lesions. Structural analysis and the following validation identify LEI110 as a potent AP-2α inhibitor. Together, we demonstrate that LEI110 stabilizes AP-2α and sensitizes HCC cells toward DNA-damaging reagents. Altogether, we identify AP-2α as a crucial transcription modulator in HCC and propose small-molecule inhibitors targeting AP-2α are a promising novel class of anticancer agents. Our study provides insights into the concept of macroscopic inhibition of DNA damage repair-related genes in cancer treatment.

## Abstract (web summary)

In hepatocellular carcinoma, TFAP2A is a key player in the transcriptional regulation of a cluster of DNA damage repair genes: DNA repair is prevented by TFAP2A depletion, and the new TFAP2A inhibitor LEI110 shows anti-tumour efficacy in HCC cell lines.

## Introduction

Hepatocellular carcinoma (HCC) is one of the most common types of liver malignancy and the leading cause of cancer-related death worldwide^1,2^. Around half of HCC cases were diagnosed in Southeast Asia, among which around 80% were observed in China^3^. However, the treatment for HCC is still limited. While multiple approaches have been suggested, including VEGFR inhibitors (such as Lenvatinib), immunotherapy (such as PD-1 inhibitor), and cell-based therapy (such as CAR-T), the overall prognosis of HCC patients is unsatisfactory^4–6^.

Oxidative DNA damage is predominantly produced in mitochondria during various physiological processes, such as proliferation, energy metabolism, responses to exogenous stimuli, etc^7^. Current knowledge of the DNA damage repair pathways, especially their modulation network, remains limited. Three major pathways were reported in the progression of HCC, including base excision repair, mismatch repair, and homologous end-joining^8–10^. However, the underlying networks controlling the expression of these DDR pathways have not been explored.

Transcription factor AP-2α (AP-2α) is a well-established transcription factor in cells^11^, responsible for various physiological and pathological processes, including nephron segment development, neural crest development, ferroptosis, and Wnt pathway, etc.^12–15^. However, the function of AP-2α in malignant tumors remains controversial: it was recognized as a predominant oncogene that promotes metastasis in multiple cancer types, including lung squamous cell carcinoma, pancreatic adenocarcinoma, and bladder cancer^16–18^. On the other hand, AP-2α was reported to induce apoptosis and suppress cell proliferation in retinoblastoma and cardiac myocytes^19,20^. Even in HCC, studies showed overexpression of AP-2α correlated with unfavorable prognoses^21,22^ and chemoresistance in patients^23^. However, AP-2a was also recognized as a tumor suppressor in HCC by suppressing the Wnt pathway^24^. Here in this study, we established the oncogenic, transcription activity of AP-2α in HCC, which specifically modulated the expression of DDR genes and promotes cancer proliferation. Together, we introduced LEI110 as a specific small-molecular inhibitor targeting TFAP2A and LEI110 is a promising novel class of anticancer agents.

## Methods

### Proliferation and clonogenic assays

#### Growth curve

3000 cells were seeded in triplicates in a 96-well plate. Cell viability was measured for 4 consecutive days with Cell Count Kit 8 and results were plotted with GraphPad Prism 9.3.1.

#### Clonogenic assay

Cells were seeded at 2000 cells/well in 6-well plates the following day and cultured in complete media (refreshed every 3 days). Colonies were counted manually after 10 days.

### Heatmap and hierarchical clustering

Heatmap with hierarchical clustering was achieved with ggplot and hclust package in the R software version 4.0.2 (2020-06-22) (https://www.r-project.org/). For Heatmap, the expression level of each gene was normalized to the median in each patient, and the color scale was normalized. Hierarchical clustering was performed with the Euclidean clustering method with complete linkage and optimized gene/sample order. For heatmaps describing the Pearson correlation between two genes, the Euclidean clustering was directly performed without the normalization. Heatmap and the following hierarchical clustering enabled us to directly visualize and comprehend the expression pattern of a certain cluster of genes or samples.

### EdU staining

Cells were seeded on a coverslip and incubated with 10 uM 5-Ethynyl-2′-deoxyuridine (EdU, Invitrogen, A10044) in culture media for 30 min before the experiment. Cells were fixed with polyformaldehyde (PFA) and permeabilized with 0.5% Triton in Phosphate Buffered Saline (PBS). Click-iT™ Cell Reaction Buffer were prepared according to the protocol: 1 mM CuSO_4_, 10 uM Alexa Fluor 595 azide, 100 mM Tris (pH 7.5), and 100 mM ascorbic acid (Sigma, A92902). After 30 min incubation with reaction buffer, cells were washed three times with PBS.

### Immunofluorescence

Cells were seeded on coverslips in 24-well plates for observation with confocal microscopy. Cells were fixed with 3.7% PFA for 10 min followed by cold methanol (−20 °C) permeabilization for 10 min. Cells were washed with PBS twice and blocked for 1 h at room temperature with the blocking solution (5% Bovine Serum Albumin [BSA] complemented with 0.1% Tween-20 in PBS). Cells were incubated with the primary antibody (γH2AX, 2577 s, CellSignalling, rabbit origin; 8-oxodG, sc-130914, Santa Cruz, mouse origin) in the blocking solution overnight. Cells were washed three times with PBS-Tween 20 (0.1%) and the secondary antibody was diluted in the blocking solution for 1 h at room temperature in the dark. Cells were washed three times with PBS-Tween 20 (0.1%). Image acquisition was then performed and results were analyzed with Cellprofiler.

### Dual luciferase assay

Dual-luciferase reporter assay was performed according to the manufacturer’s protocol (Beyotime, RG027). Briefly, cells were co-transfected with firefly luciferase control plasmid along with renilla reporter plasmid at a ratio of 10:1. After 48 h, cells were harvested and lysed in the lysis buffer. The activity of luciferase was detected by the Dual-Luciferase Reporter Assay System. The results were normalized to the renilla activities.

### Motif finding and binding prediction at the promoter region

Mutual domains at the promoter regions of different genes were analyzed using the GLAM2 (Gapped Local Alignment of Motifs) tool of MEME Suite (Multiple Expectation Maximization for Motif Elicitation suite, https://meme-suite.org/meme/tools/glam2). The transcription binding motifs on the mutual domains of these genes were predicted.

For the prediction of the binding site of AP-2α on the promoters of selected genes: promoter regions (2000 bp) were acquired at the UCSC genome browser; then, JASPAR (http://jaspar.genereg.net/) and PROMO (https://alggen.lsi.upc.es/cgi-bin/promo_v3/promo/promoinit.cgi?dirDB=TF_8.3) websites were used to determine the binding sites of AP-2α.

### Cell lines and cell culture

Human HCC cell lines HEP3B and SNU387 were purchased from ATCC. Human HCC cell lines Huh7 and MHCC-97H were purchased from the National Collection of Authenticated Cell Cultures (https://www.cellbank.org.cn/). HEP3B cells were cultured in Minimum Essential Media (MEM, Gibco, 11095080) Supplementaryemented with 1% Non-Essential Amino Acids (NEAA, Sigma Aldrich, M7145) and 10% fetal bovine serum (FBS, Gibco, 12662029). Huh7, MHCC-97H, and SNU387 cells were cultured in Dulbecco’s Modified Eagle Medium (DMEM, Gibco, 11995065) with 10% fetal bovine serum (FBS, Gibco, 12662029). All cells were cultured in a humidified incubator containing 5% CO_2_ at 37 °C.

### siRNA transfections

HEP3B and Huh7 cells were seeded in 6-well polystyrene microplates and incubated until they reached 30–40% confluence. Cells were transiently transfected with siRNA at a final concentration of 10 nM with INTERFERin (#409-01, Polyplus) transfection reagent according to the manufacturer’s instructions.

siRNA sequences used are listed below^25,26^:

siAP-2α sequence 1: CCUGCUCACAUCACUAGUATT

siAP-2α sequence 2: GGGUAUUAACAUCCCAGUTT

### Cellular thermal shift assay (CETSA) assay

CETSA assay was carried out as previously described in ref. ^27^. Briefly, cells were seeded in a 15 cm dish and cultured until 80–90% confluency. Cells were treated with DMSO or LEI110 for 1 h before the experiment. Cells were then resuspended and washed twice with PBS. Cells were aliquoted in 7 PCR tubes and heated at increasing temperatures for 3 min. Cells were snap-frozen in liquid nitrogen and thawed at 25 °C twice. Samples were then lysed using RIPA buffer, centrifuged and the lysate was collected and stored at −80 °C, until further analysis by western blot. Original figures for the western blot were Supplied in the Supplementary material (Supplementary Fig. 8).

### Inhibitors and reagents

The following inhibitors and reagents were used: H2DCFDA was purchased from MedChemExpress (HY-D0940), CCK8 kit was purchased from MedChemExpress (HY-K0301), Cisplatin was purchased from Selleck (S1166), hydroxyurea was purchased from MERK (H8627) and TH5487 was purchased from MedChemExpress (HY-125276). Hoechst 33258 for nuclear staining was purchased from MERK (94403).

### Chromatin Immunoprecipitation (ChIP)-qPCR

ChIP was performed with a Chromatin Immunoprecipitation kit (Merck Millipore, MA, USA) per the manufacturer’s instructions. Briefly, cells were cultured in a 15 cm dish till 80% confluency and were crosslinked with 550 ul 37% formaldehyde (1% final concentration) for 10 min at room temperature. Cells were harvested and the nuclear was isolated and resuspended in SCW buffer (Merck Millipore, 17-10460). After sonication on ice, samples were centrifuged and the supernatant was collected. At the same time, Magna ChIP Protein A/G Magnetic Beads were washed and incubated with AP-2α antibody (Proteintech, 13019-3-AP) or IgG control. Then samples were immunoprecipitated with antibody-labeled Magnetic Beads overnight. Samples were repeatedly washed and incubated with Elution Buffer with proteinase K.

Immunoprecipitated DNA was collected and the enrichment of the DNA template was analyzed by conventional quantitative PCR, using primers specific for each gene. qPCR was performed as described in the Supplementary material and primers used are listed as below:

Target name Sequence Target size

ChIP_TOP2A_F: 5’-AAACGAAGCTAAGGCTCCCA-3’ 188

ChIP_TOP2A_R: 5’-GTGCGGAAAGCTTGGAAGAG-3’

ChIP_NUDT1_F: 5’-CGGGCAATGGAGCTACCC-3’ 192

ChIP_NUDT1_R: 5’-GGATTCCGGGTCGCAGTTC-3’

ChIP_PARP1_F: 5’-CACCTCGGGCCAATCAACTA-3’ 82

ChIP_PARP1_R: 5’-TGTGTCCTCTCTCCCCTGAG-3’

ChIP_POLD1_F: 5’-CAGAGGCCTCAGCCTCAGGGT-3’ 111

ChIP_POLD1_R: 5’-TTGTTCGGACAGAAGTCCAGG-3’

### Virtual screen and molecular dynamic simulation

#### Protein preparation

PDB files (8J0L^28^ for human AP-2α and A0A286YD43 for mouse AP-2α) were downloaded from Alphafold2 (https://www.alphafold.ebi.ac.uk/) imported to Chimera version 1.17.3^29^(https://www.cgl.ucsf.edu/chimera/download.html) and performed high throughput screening for the AP-2a. In brief, Dock Prep was performed in the AP-2a protein: bond orders were assigned, hydrogens were added, disulphide bonds were generated. Afterwards, charges were added and Gasteiger was applied for other residues and Net Charges were specified. And the grid was generated, covering the whole TFAP2A protein. Then the non-covalent docking between protein and ligands were performed using the Autodock Vina implanted in the Chimera^30,31^.

#### Ligand preparation

ligands were prepared in Chimera using the Dock prep function, the same as the protein preparation process. Briefly, the ligand conformations were energy minimized using MOPAC^32^ before seeding the conformation in Autodock Vina. The solvent and non-complexed ions were removed, hydrogen and charges were added, and the topological information (rotatable bonds) was acquired. Then the PDBQT file was exported for the ligand before the docking experiment^33^. But the tautomerization was not considered in this screen.

Ligand database used for virtual screen was provided by TargetMol (https://www.targetmol.cn/) or ZINC database (https://zinc.docking.org/) and 3 poses were calculated for each compound.

Further, the binding of small molecules at the active site in TFAP2A was manually selected, as previously reported^34^.

Molecular dynamics: molecular dynamics studies were performed with Desmond as implemented in Schrödinger^35^. Briefly, the system was set up by merging the protein into a standard orthorhombic box in SPC (original and refined) water. The system was neutralized by adding Chloride ions and 0.15 NaCl was added. The OPLS2005 force field was used and molecular dynamics simulation was performed using the generated system. A 100 ns of simulation was calculated in 310 K, 1.01325 bar, and the results were analyzed in Schrodinger.

### Modified Comet assay

Cells were suspended in 0.5% low melting point agarose in PBS and transferred onto a frosted glass microscope slide precoated with a layer of 0.5% normal melting point agarose. Slides were immersed in lysis solution at 4 °C overnight. Cells were washed with enzyme assay buffer and incubated with FpG enzyme (#M0240S, New England Biolabs) in enzyme assay buffer or buffer alone for 30 min at 37 °C. Electrophoresis buffer was precooled to 4 °C and slides were incubated in electrophoresis buffer for 30 min. Electrophoresis was run at 300 mA, 25 V for 30 min. Slides were washed in neutralization buffer and counterstained with SYBR GOLD. At least 50 comets per sample were analyzed.

### Synergetic experiments

Synergy experiments were performed in a 96-well plate. Cells were treated with increasing concentrations of LEI110 and other inhibitors/reagents, incubated for 72 h and cell viability was measured with CCK8. Synergy score was calculated with SynergyFinder (http://synergyfinder.fimm.fi/) using ZIP method.

### Gene Set Enrichment Analysis (GSEA)

GSEA was performed using the GSEA 4.0.3 software. Gene sets for gene ontology_biological process and KEGG analysis were downloaded for the analysis and results were permutated according to phenotype and repeated for 1000 times.

### Cell cycle expression profile for DDR genes

A database for cell cycle-dependent, single-cell sequencing was described in GSE146773. A FUCCI transfected U2OS cells were used and the expression profile of DNA damage repair-related genes were acquired in different cell cycles. And G1, G1/S, G2, and Mitosis phases were shown (Supplementary Fig. 3b).

### Statistics and Reproducibility

mRNA sequencing data (gene expression RNAseq - IlluminaHiSeq pancan normalized data, *n* = 423), CNV data (*n* = 372), and clinical data for tumor tissue and non-tumor tissue (Curated survival data and phenotypes, n = 438) were acquired from the TCGA LIHC database (https://tcga-data.nci.nih.gov/).

**Fig. 1:**
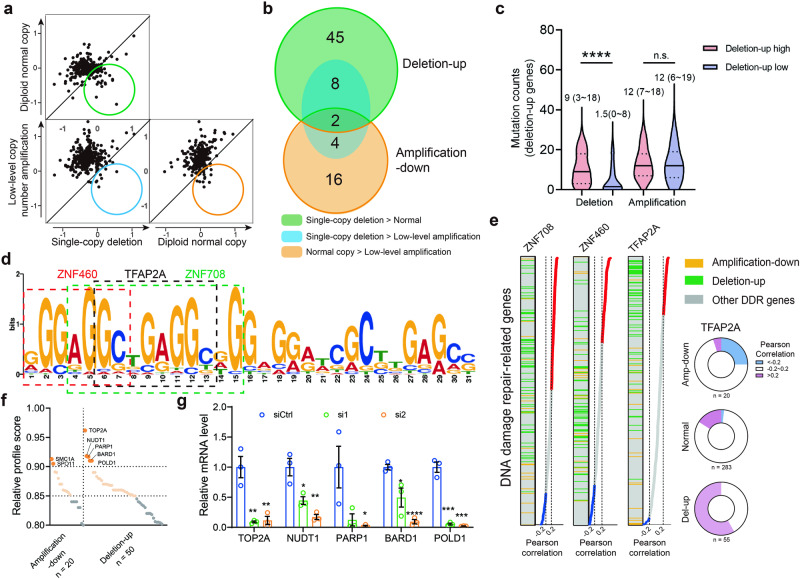
AP-2α modulates the expression of "deletion-up" genes in HCC patients. **a** Relative expression levels of DDR-related genes in HCC patients with different CNV. 3 major types of CNV were included: single-copy deletion, diploid normal copy, and low-level amplification. **b** Venn plot showing the distribution of amplification-down genes and deletion-up genes defined in HCC patients. **c** Violin plot showing the somatic mutation frequency of deletion-up genes in HCC patients. The median and the 25%, 75% percentile were labeled and shown in the solid and dotted line respectively. **d** Top common motifs observed in the deletion-up genes in HCC patients. The binding of transcription factors was predicted and labeled. **e** Correlation between the expression levels of different DDR genes and 3 predicted transcription factors: ZNF708, ZNF460, and AP-2α. Lower right panel: pie chart showing the percent of DDR genes with strong correlation to AP-2α. **f** Dot plot showing the predicted binding affinity of AP-2α on the promoter region of deletion-up genes in JASPAR. **g** Relative mRNA levels of *TOP2A, NUDT1, PARP1, BARD1*, and *POLD1* in normal or AP-2α-depleted HEP3B cells. *n* = 3 biologically independent samples. Students’ *t*-test, *p < 0.05, **p < 0.01, ***p < 0.001, ****p < 0.0001. Means ± SEM from three independent experiments.

**Fig. 2:**
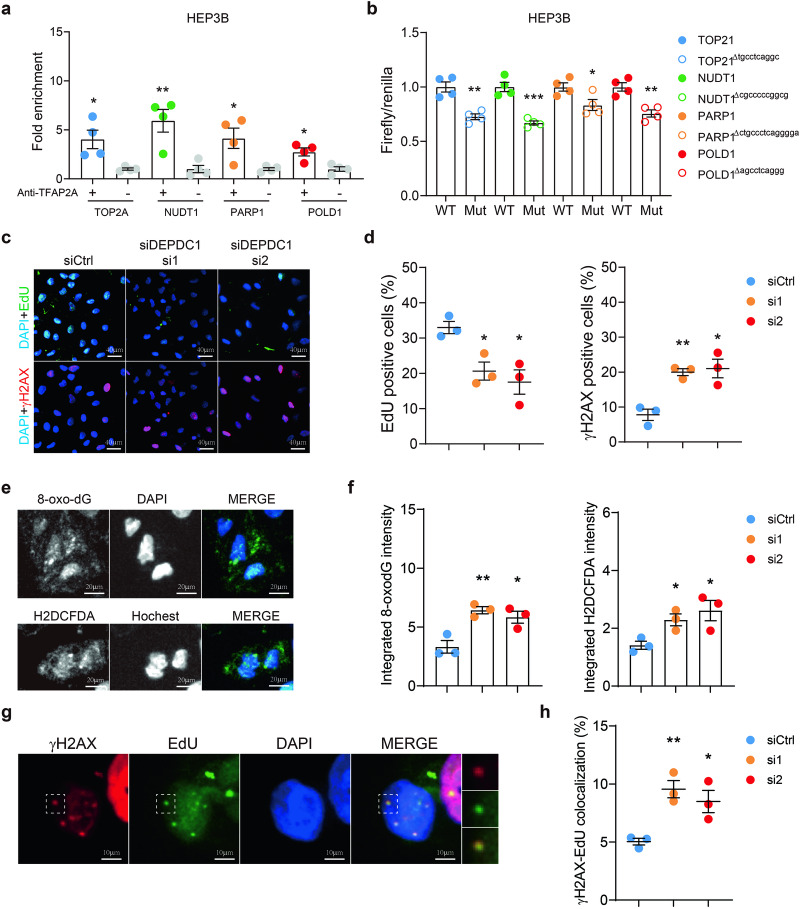
AP-2α transcriptionally moulates DNA damage repair in HCC. **a** Quantification of chromatin immunoprecipitation (ChIP)-qPCR results in HEP3B cells. The binding of *TOP2A, NUDT1, PARP1*, and *POLD1* was measured. *n* = 3 biologically independent samples. **b** Quantification of dual luciferase assay data in HEP3B cells. Cells were transfected with wild-type or mutated firefly constructs of *TOP2A, NUDT1, PARP1*, or *POLD1* promoter regions, respectively. Renilla plasmid was co-transfected as the control of the transcription efficacy. *n* = 3 biologically independent samples. **c**, **d** Typical figures (**c**) and quantification of EdU staining and γH2AX staining in normal or AP-2α-depleted HEP3B cells. *n* = 3 biologically independent samples. **e**, **f** Typical figures (**d**) and quantification of 8-oxo-dG staining and H2DCFDA signal in normal or AP-2α depleted HEP3B cells. *n* = 3 biologically independent samples. **g**, **h** Typical figures (**g**) and quantification of EdU-γH2AX colocalization in normal or AP-2α-depleted HEP3B cells. *n* = 3 biologically independent samples. Students’ t test, *p < 0.05, **p < 0.01, ***p < 0.001. Means ± SEM from three independent experiments.

**Fig. 3:**
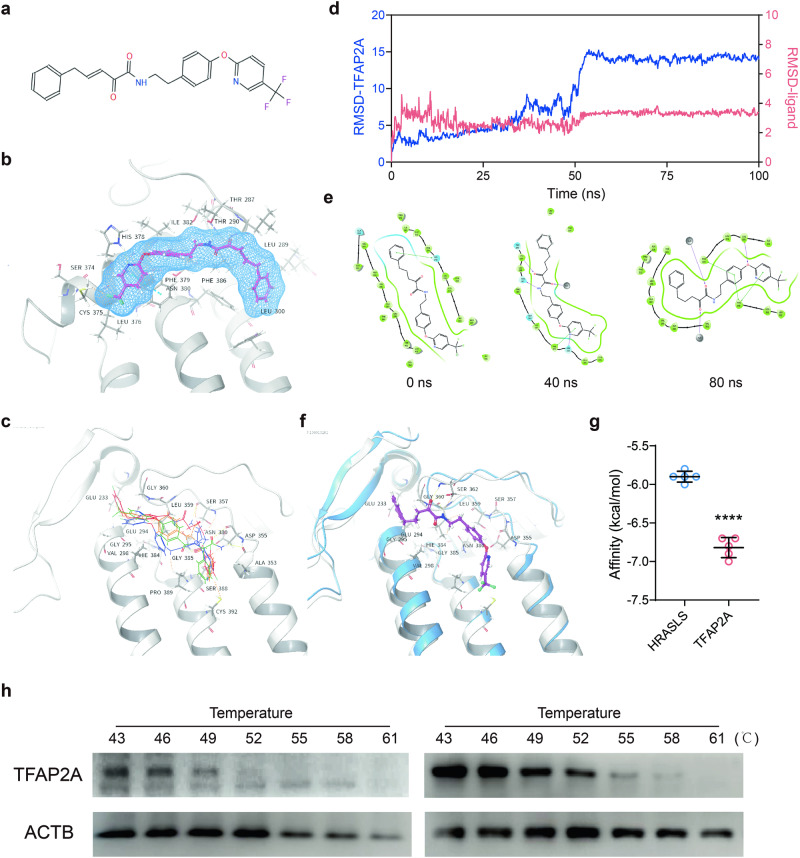
LEI110 is a small-molecular inhibitor for AP-2α. **a** The chemical structure of LEI110. **b**, **c** The binding of LEI110 (**b**) and similar structures (**c**) with AP-2α in Chimera. Residues adjacent to the binding site were labeled. **d**, **e** Quantification (**d**) and typical ligand-receptor interaction figures at 0, 40 ns, and 80 ns (**e**) of one typical molecular dynamic simulation result using the Desmond. **f** Aligned binding of human AP-2α (8J0L in the PDB, shown in while) and mouse AP-2α (A0A286YD43 in Alphafold, shown in blue) with LEI110. **g** Quantification of docking score showing the binding affinity (kcal/mol) between HRSLS-LEI110 and AP-2α-LEI110. The top 5 poses were included for each group. (**h**) CETSA experiment showing the stabilization of AP-2α by LEI110 at different temperatures. Students’ *t*-test, ****p < 0.0001. Means ± SEM from three independent experiments.

**Fig. 4:**
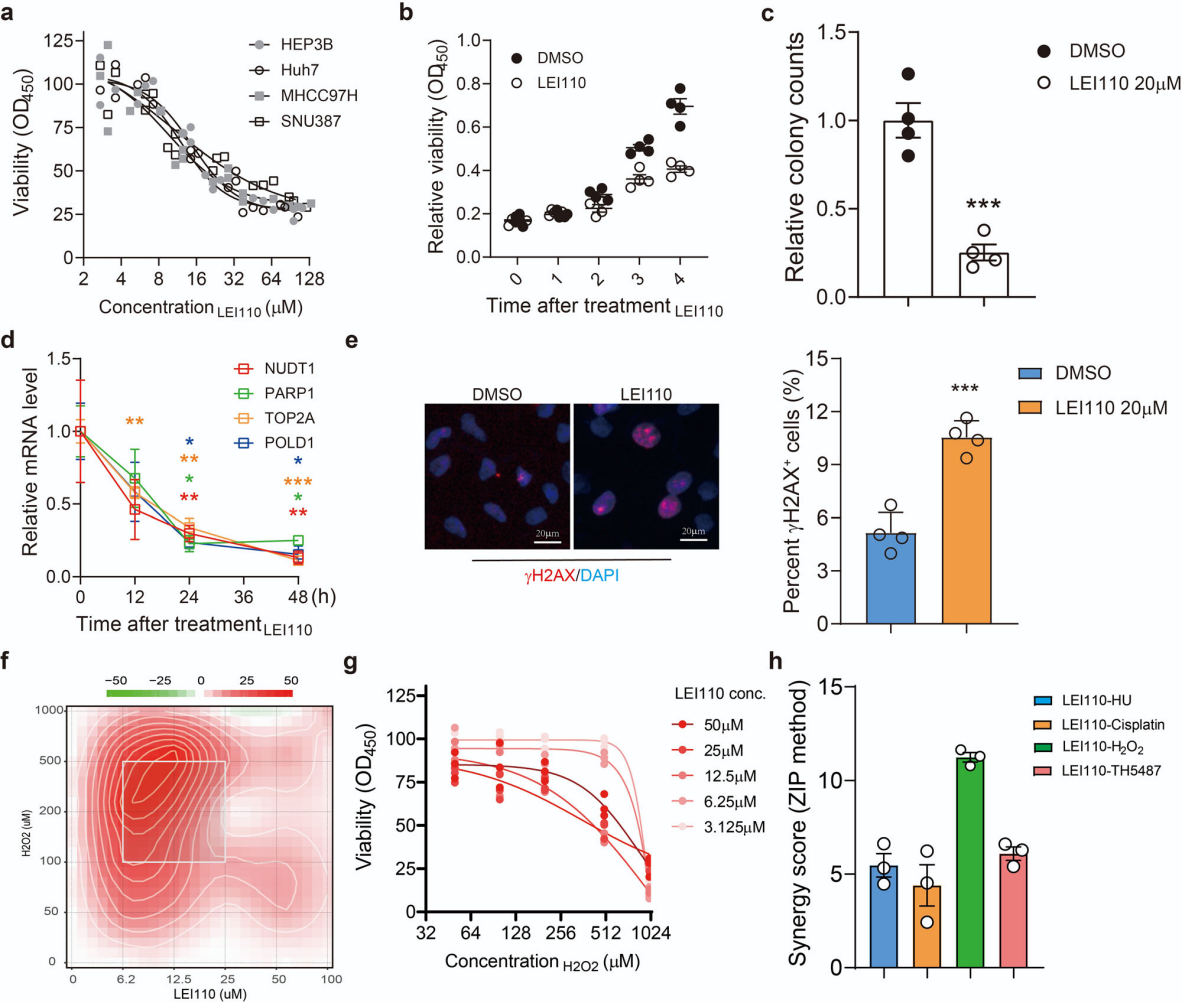
LEI110 eradicates HCC by inducing DNA damage in cells. **a** Dose-response result showing the inhibition of 4 HCC cell lines by LEI110. *n* = 3 biologically independent samples. **b**, **c** Quantification of proliferation ability (**b**) and clonogenic ability (**c**) in HEP3B cells after LEI110 treatment. *n* = 3 biologically independent samples. **d** Quantification of TOP1, NUDT1, PARP1, and POLD1 mRNA levels after LEI110 treatment at 0 h, ‘12 h, 24 h and 48 h. *n* = 3 biologically independent samples. **e** Typical figures (the left panel) and quantification (the right panel) of γH2AX foci in HEP3B cells treated with LEI110. *n* = 3 biologically independent samples. **f**, **g** Synergy plot (**f**) and dose-response of H_2_O_2_ (**g**) in HEP3B cells treated with increasing levels of LEI110. *n* = 3 biologically independent samples. **h** Quantification of synergy scores (ZIP method) between LEI110 and 4 different DNA damage-inducing reagents: H_2_O_2_, Cisplatin, TH5487 and hydroxyurea. *n* = 3 biologically independent samples. Students’ *t*-test, *p < 0.05, **p < 0.01, ***p < 0.001. Means ± SEM from three independent experiments.

Correlation between two genes in HCC patients was performed with SPSS statistics 26 (Supplementary Fig. 1a, c). Kaplan-Meier analysis and student’s t-test were carried out with GraphPad Prism 9.3.1, including the results in Figs. 1g, 2a, b, d, f, h, 3g, 4c–e, and in Supplementary material Supplementary Fig. 1d, Supplementary Fig. 4a, b, d, Supplementary Fig. 6b.

Dose-response results were simulated using the [Inhibitor]-response – variable slope (four parameters) in the GraphPad Prism 9.3.1 (Fig. 4a, g, Supplementary Fig. 6d, f, h).

Kaplan Meier analysis was performed using GraphPad Prism 9.3.1 for the overall survival and progression-free interval (PFI) in Supplementary Fig. 1h and Supplementary Fig. 2e. Patients were divided into high and low-expression groups according to the median expression level.

### Quantitative real-time polymerase chain reaction

Real-time polymerase chain reaction (RT-PCR) was performed using SYBR Premix Ex Taq (Takara) in an ABI PRISM 7900HT sequence detector. annealing and reading temperatures are 95 °C and 55 °C respectively. Two housekeeping genes, b-Actin, and GAPDH, were used as endogenous controls and the primer sequences used in this paper are as follows:

Gene Sequence Product size

*TOP2A_F* 5’-ACCATTGCAGCCTGTAAATGA-3’ 129

*TOP2A_R* 5’-GGGCGGAGCAAAATATGTTCC-3’

*NUDT1_F* 5’-GCTCATGGACGTGCATGTCTT-3’ 140

*NUDT1_R* 5’-GTGGAAACCAGTAGCTGTCGT-3’

*PARP1_F* 5’-CGGAGTCTTCGGATAAGCTCT-3’ 136

*PARP1_R* 5’-TTTCCATCAAACATGGGCGAC-3’

*POLD1_F* 5’-AGCAGGTCAAGGTCGTATCC-3’ 211

*POLD1_R* 5’-AGCGTGGTGTAACACAGGTTG-3’

*BARD1_F* 5’-GGTATCCTTCTGTAGCCAACCA-3’ 79

*BARD1_R* 5’-GGAGCCACTTGCTAGTAAGTCT-3’

*ACTB_F* 5’-CATGTACGTTGCTATCCAGGC-3’ 250

*ACTB_R* 5’-CTCCTTAATGTCACGCACGAT-3’

### Reporting summary

Further information on research design is available in the Nature Portfolio Reporting Summary linked to this article.

## Results

### The characterization of deletion-up genes in HCC

CNV and transcription factor activity profoundly affect the abundancy of mRNA in cells. To understand the modulation network of DDR genes depending on transcription factors, rather than the CNV levels, we focused on a cluster of genes overexpressed in patients with single-copy deletion (“deletion-up” genes) or genes down-regulated in patients with DNA copy amplification (“amplification-down” genes).

Briefly, the CNV levels (including single copy deletion, normal copy, and low-level copy number amplification) and their corresponding mRNA expression levels of all 360 DDR-related genes^36,37^ in the TCGA LIHC database was collected (Supplementary Fig. 1a). The expression of DDR gene with respect to different CNV levels were quantified and a cluster of genes whose expression were higher in patients with single-copy deletion, compared to patients with diploid/low-level amplification, were identified as “deletion-up” genes (Fig. 1a and Supplementary Fig. 1b). Similarly, a cluster of low-level amplification genes was defined as “amplification-down” genes (Fig. 1a and Supplementary Fig. 1b). The distribution and overlaps of “deletion-up” genes, such as *TOP2A, NUDT1, PARP1*, and *POLD1, and “amplification-down” genes, such as SMC1A and SPO11, were plotted in* Fig. 1b, c and we deduced that the expression of these DNA damage-related genes was predominantly modulated by the activity of transcription factors.

Mutation counts of deletion or amplification were quantified in “deletion-up” genes and “amplification-down” genes and clustered with the Euclidean method (Supplementary Fig. 1c, d). Consistent with previous studies, the expression of DDR genes was coordinately expressed in HCC, yet the CNV levels were not^37,38^. Most deletion-up genes were simultaneously co-expressed in HCC patients, independent of the CNV levels (Supplementary Fig. 1c, e). More importantly, the synergistically overexpressed “deletion-up” genes correlated with multiple activated DNA damage repair pathways and decreased overall survival and the progression-free interval of HCC patients (Supplementary Fig. 1f–h).

### AP-2α promotes replication and DNA damage repair by transcriptionally modulating DDR gene expression

Mutual gene sequences of the “deletion-up” genes and “amplification-down” genes were aligned and analyzed with the MEME. Further, we predicted the binding of all the transcription factors on the mutual promoter sequences in deletion-up genes and amplification-down genes, among which *ZNF708, AP-2α*, and *ZNF460* were observed as the top hits for deletion-up genes (Fig. 1d–f, Supplementary Fig. 2a–d, g, h). AP-2α preferentially correlates with deletion-up genes and patients with dysregulated AP-2α or “deletion-up” genes showed unfavorable prognosis (Fig. 1e, Supplementary Fig. 2e, f). Patients with high AP-2α levels in HCC also showed obvious activation of multiple DNA replication and DDR pathways (Supplementary Fig. 1g).

With chromatin immunoprecipitation (ChIP-qPCR), the binding of AP-2α on the promoter regions of 4 major DNA damage repair genes, *TOP2A, NUDT1, PARP1, BARD1*, and *POLD1*, was established (Fig. 2a, Supplementary Fig. 3a). The transcription efficacy of these genes was demonstrated with the dual luciferase assay (Fig. 2b, Supplementary Fig. 3b).

Most “deletion-up” genes were enriched in the G2 and M phases (Supplementary Fig. 3c), and the G2/M transition and replication pathways were enriched in HCC patients with high AP-2α expression, suggesting the involvement of AP-2α-DDR axis in the DNA replication and damage repair processes (Supplementary Fig. 3a, b). In AP-2α-depleted HEP3B cells, decreased replication (EdU-positive cells) and elevated DNA replication damage (γH2AX foci and EdU-γH2AX colocalization) were observed (Fig. 2c, d, g, h, Supplementary Fig. 4c, d). Also, elevated 8-oxo-dG and ROS levels (with H2DCFDA staining) were observed in AP-2α-depleted HEP3B cells and Huh7 cells (Fig. 2e, f).

### LEI110 was a potential AP-2α inhibitor and eradicates HCC by inducing oxidative DNA damage

Currently, no potent AP-2α inhibitors have been reported. In the pursuit of AP-2α inhibitors, we screened a total of > 200,000 bioactive small molecules in the TargetMol database and expanded the top hits with similar structures in the ZINC database. LEI110 and its analogs were identified as potential inhibitors for both human and mice AP-2α protein (Fig. 3a–c, Supplementary Fig. 5a). Molecular dynamics assay showed effective stabilization of both human and mouse AP-2α by LEI110 (one typical MD results were shown in Fig. 3d–f, Supplementary Fig. 5b–d). Also, the stabilization was weakened after 50 ns in human AP-2α and gradually weakened in mouse Tfap2a, suggesting LEI110 was a moderate binder of AP-2α. As we noticed, while LEI110 was trapped in the binding pocket, the interaction pattern between LEI110 and AP-2α varied within 100 ns (Fig. 3d, e, Supplementary Fig. 5b). Therefore, it could be deduced that LEI110 is a flexible, weak-to-moderate binder of AP-2α. And this could be due to the mismatch between LEI110 and the spacious binding pocket of AP-2α.

LEI110 was previously reported as a selective pan-inhibitor of the HRASLS family of thiol hydrolases (i.e., PLA2G16, HRASLS2, RARRES3, and iNAT)^39^. Yet a stronger binding was observed in LEI110 with AP-2α (8J0L)^28^, compared with PLA2G16 (4Q95^40^) in terms of docking scores in Chimera (Autodock Vina) and for molecular dynamics in the Desmond of Schrodinger (Fig. 3g, Supplementary Fig. 5g). Of note, the molecular dynamics for LEI110-AP-2a have been repeated several times with similar trends and only the typical results were shown in Fig. 3g. Consistently, the stabilization of AP-2α by LEI110 was observed in the CETSA, validating the binding of LEI110 with AP-2α (Fig. 3h).

The cytotoxicity of LEI110 was tested in a dose-response assay and the result showed LEI110 efficiently eradicated HCC cell lines (Fig. 4a). Similarly, LEI110 could suppress the proliferation and clonogenic ability of HCC cells, which was rescued by exogeneous expression of AP-2α (Fig. 4b, c, Supplementary Fig. 6a, b). Also, we performed the modified comet assay in HEP3B cells after LEI110 treatment to measure the oxidized DNA lesions recognized and processed by FpG. We observed an elevated tail moments after LEI110 treatment, compared with DMSO control group, suggesting the induction of oxidized DNA damage in cells (Supplementary Fig. 7a).

As expected, the expression of AP-2α was not significantly reduced after LEI110 treatment for 48 h, but its DNA binding activity was impaired (Supplementary Fig. 7b, c). LEI110 treatment transcriptionally suppressed *NUDT1, PARP1, TOP2A*, and *POLD1* expression at 24 h–48 h (Fig. 4d) and induced the accumulation of oxidized DNA lesions in HCC cells (Fig. 4e). Besides, a strong synergetic effect between LEI110 and multiple DNA damage-inducing reagents, such as hydroperoxide, hydroxyurea, OGG1 inhibitor (TH5487), and Cisplatin was observed (Fig. 4f–h, Supplementary Fig. 6c–h).

Lastly, we interrogated the toxicity of LEI110 in the liver. We gave a single dose of LEI110 to the mice at 10 mg/kg, i.p. for 24 h and evaluated the liver damage with hematoxylin-eosin staining (Supplementary Fig. 7d). As a result, no behavioral or histological alterations were observed in the mice liver, suggesting no obvious acute toxicity of LEI110 in the liver (Supplementary Fig. 7d).

## Discussion

Hepatocellular carcinoma (HCC) is the most common type of malignant tumor in the liver and the treatment for HCC is limited^1–3,6^. The urge for the development of novel targets and drugs for HCC remains intense.

Oxidative lesions are common side-products of various energy metabolism in mitochondria and DDR pathways are of great significance for the maintenance of the physiological function in cells^7,8,10^. Meanwhile, somatic mutations in HCC patients, especially for DDR-related genes, have been widely reported^41^. Therefore, understanding the mechanisms that guarantee the fast proliferation of HCC cells is very important.

We initially focused on the somatic mutation and the expression levels of DDR-related genes in HCC patients and observed that the expression levels of DDR genes correlated with its CNV levels in HCC patients. However, the expression levels of a cluster of DDR genes were independent of its CNV levels: the expression of these genes was even higher in HCC patients with single-copy deletion, compared with patients with diploid normal-copy or low-level amplification, defined as “deletion-up” genes. Similarly, the expression of a cluster of genes was lower in HCC patients with low-level amplification, compared with patients with diploid normal-copy, defined as “amplification-low” genes.

The transcription of “deletion-up” genes was predominantly dependent on the transcription status of their promoter regions. To decipher the transcription modulation network of these genes, we predicted the top mutual binding motif of these deletion-up genes and observed the binding of AP-2α (Fig. 1 and Supplementary Fig. 2A–C, Supplementary Fig. 3a, b). Consistent with previous predictions, the transcription modulation function of AP-2α was validated with ChIP-qPCR and dual luciferase assay. Specifically, AP-2α depletion effectively prevented the repair of DNA damage in HCC cells (Fig. 1 and Supplementary Fig. 4). These results suggested the rationale for AP-2α inhibition for the treatment of HCC.

Virtual screening showed that LEI110 and its analogs as potential inhibitors for AP-2α. Molecular dynamic simulation unveiled the weak-to-moderate binding affinity of LEI110 with AP-2α. While we also recognized that the comparison of the docking scores obtained for the same compound on different targets (Fig. 3g) might provide unwanted inaccuracies, possibly deriving from intrinsic structural factors related to the complexes. Therefore, further explorations would be beneficial to determine the binding preference of LEI110 in PLA2G16 and AP-2α. The following CETSA assay validated the effective stabilization of AP-2α with LEI110 in HEP3B cells (Fig. 2 and Supplementary Fig. 5) and the tumor-suppressing function of LEI110 was observed in HCC cell lines. Besides, LEI110 not only induced the accumulation of DNA damage and the suppressed expression of multiple DDR genes, but it also sensitized HCC cells towards multiple DNA damage-inducing reagents, such as hydroperoxide, hydroxyurea, OGG1 inhibitor (TH5487), and Cisplatin (Fig. 2 and Supplementary Fig. 6).

In this study, we proposed the overexpression of a cluster of “deletion-up” genes in HCC, which were transcriptionally modulated by TFAP2A (AP-2α). While AP-2α is an intriguing target due to its controversial effects in different cancers and the complete abolishment of AP-2α might be toxic in vivo. However, we still believe that the inhibition of AP-2α with small-molecule inhibitors would be a promising direction for the future, especially for cancers with AP-2α overexpression. The advantage is that we can suppress the expression of multiple oncogenes with AP-2α inhibition and the toxicity of the drug could be avoided by adjusting the dosage of the drug. Furthermore, while transcription factors affect the expression of various targets, the expression of each gene is modulated by many transcription factors, and therefore the toxicity can be somehow avoided by lowering the dosage. Altogether, we believe AP-2α is a promising target for cancer management and LEI110, together with its analogues, is a prospective entity for AP-2α inhibition worth exploring.

## Conclusion

Together, our HCC-related findings defined a cluster of deletion-up DDR genes, the expression of which was predominantly modulated by their mutual transcription factor, AP-2α. More importantly, we identified LEI110 as an effective, unrecognized inhibitor of AP-2α and established the anti-tumor activity of LEI110 in HCC cells. Furthermore, LEI110 was shown to sensitize HCC cells towards multiple DNA damage-inducing drugs, which provided potential clinical insights into HCC treatment. Although the majority of our study was performed in vitro, we believe our data would potentially benefit the basic and clinical investigation of HCC in the short future.

### Supplementary information

Supplementary Figs.

Description of Additional Supplementary Files

Supplementary Data 1

Reporting Summary

## Data Availability

The datasets generated and/or analyzed during the current study are available in the TCGA database at https://portal.gdc.cancer.gov/projects/TCGA-LIHC. The structure for TFAP2A was available at AlphaFold (https://www.alphafold.ebi.ac.uk/) under the accessionA0A286YD43 for mouse AP-2α. And the structure for AP-2α in human was acquired from PDB database under the accession 8J0L^28^. The numerical source data for graphs and charts in the manuscript was uploaded as Supplementary data 1. The uncropped figures for western blot experiments were demonstrated in Supplementary Fig. 8. Other related data are available from the corresponding authors on reasonable request.
