# Supplementary material for: Macroscopic inhibition of DNA damage repair pathways by targeting AP-2α with LEI110 eradicates hepatocellular carcinoma
Source: Commun Biol. 2024 Mar 19;7:342. doi: 10.1038/s42003-024-05939-7 (PMC10951303; doi:10.1038/s42003-024-05939-7)
Supplement: Supplementary file 1 — Supplementary Figs. [file 42003_2024_5939_MOESM1_ESM.pdf]

Supplementary Figures

Suppl. Figure1

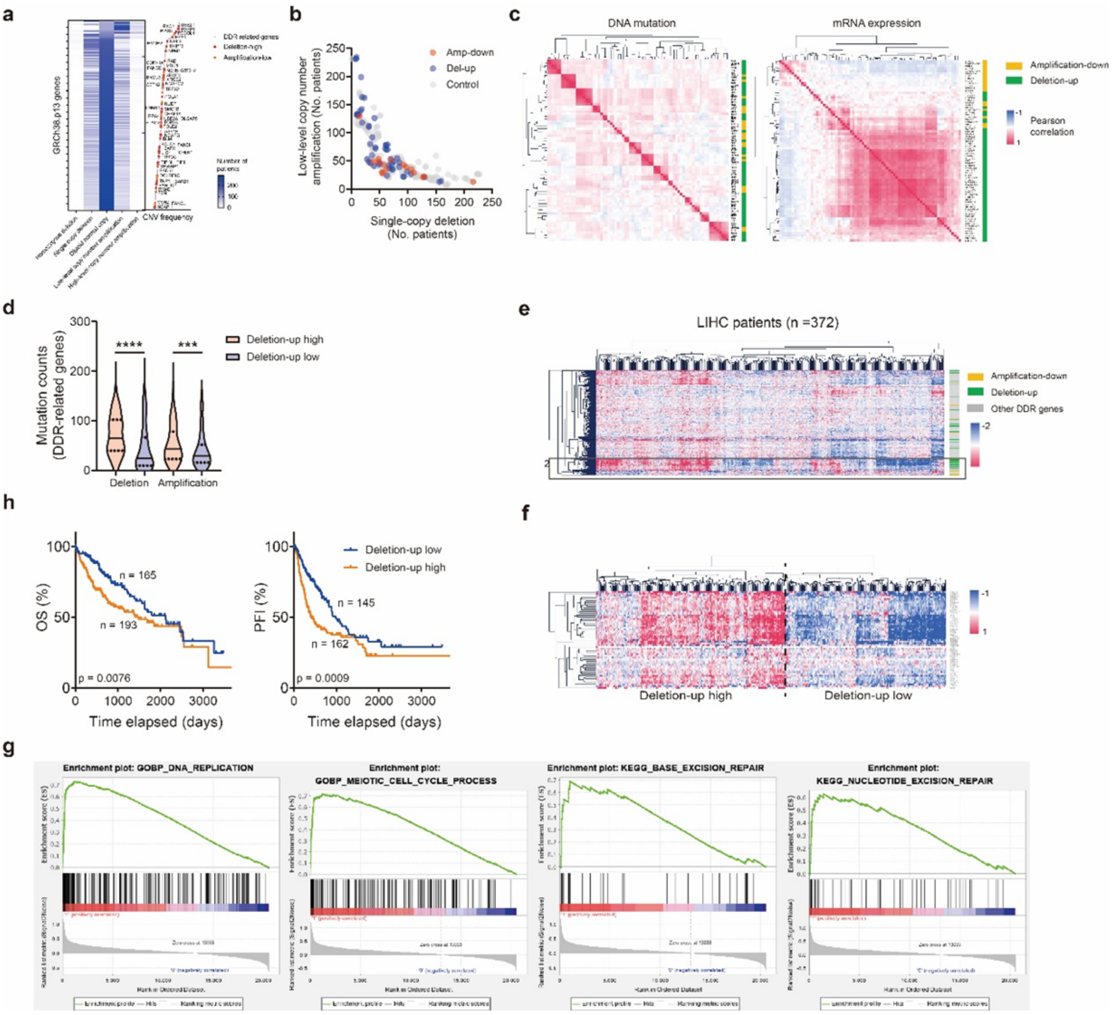

(A-B) Dot plot showing the distribution of SNV levels in HCC patients (A) and deletion-up genes (B).

(C) Heatmap showing the DNA levels (the left panel) and mRNA expression levels (the right panel) correlation between DNA damage repair-related genes.

(D) Violin plot showing the mutation counts of DDR-related genes in HCC patients.

(E-F) Heatmap showing the expression pattern of all DDR genes (E) and deletion-up genes (F) in the TCGA LIHC database.

(H) Kaplan-Meier analysis showing the unfavorable prognosis of HCC patients with different levels of deletion-up genes.

(G) GSEA analysis results showing the enrichment of DDR pathways in patients overexpressing deletion-up genes.

Suppl. Figure2

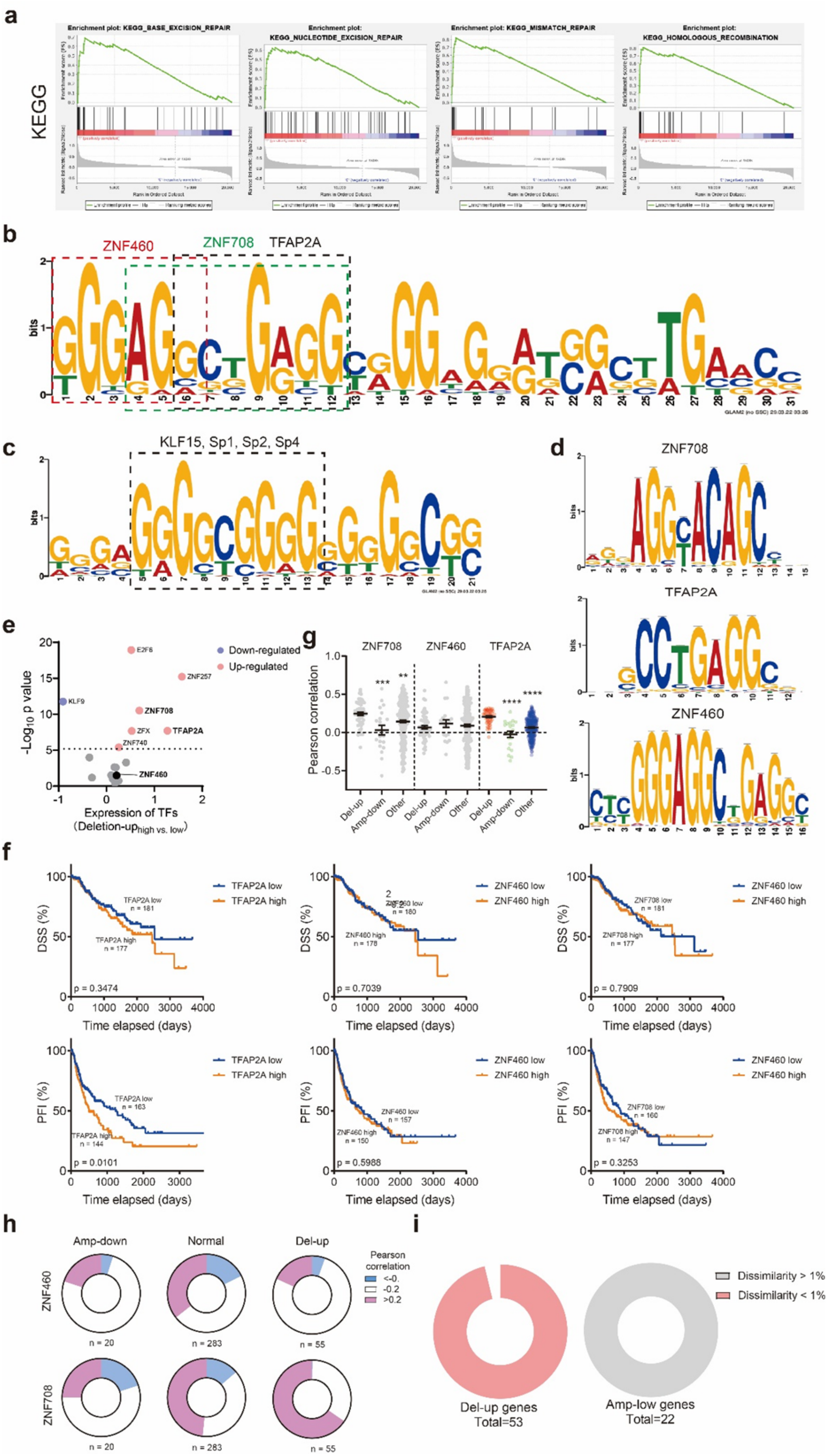

(A-B) Top mutual motifs found with the MEME website in deletion-up/amplification genes (A) or amplification-down (B) genes.
(C) Binding motifs of ZNF708, TFAP2A, and ZNF460.
(D) Expression levels of different transcription factors in the TCGA LIHC database.
(E) Kaplan-Meier analysis comparing the prognosis of patients with different ZNF708, TFAP2A, and ZNF460 levels.
(F) Dot plot showing the expression levels of ZNF708, TFAP2A and ZNF460 in HCC patients with different CNV levels.
(G) Pie chart showing the percent of genes whose expression correlated with ZNF708, TFAP2A, and ZNF460 in the TCGA LIHC database.
(H) Pie chart showing the TFAP2A binding prediction results on the promoter regions of deletion-up genes or amplification-down genes.

Suppl. Figure 3

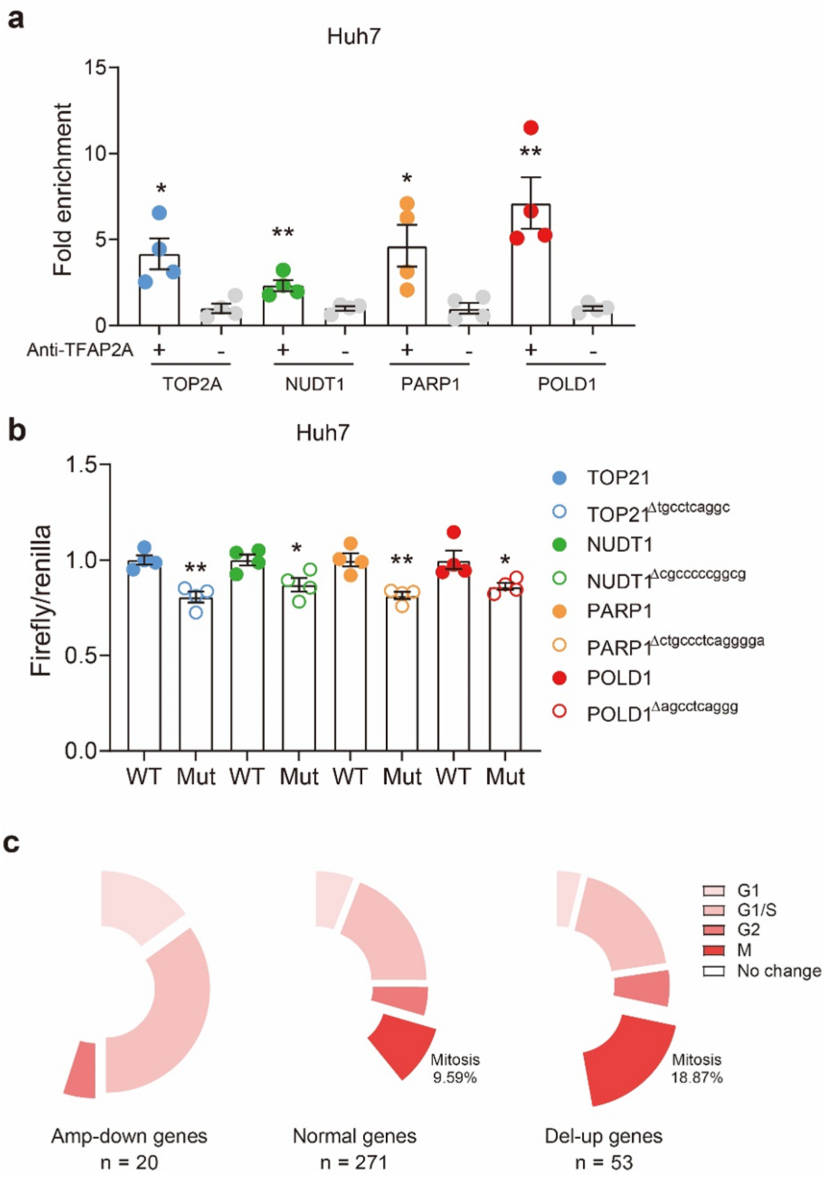

(A) Quantification of ChIP-qPCR results, showing the binding of TFAP2A on the promoter regions of TOP2A, PARP1, POLD1 and NUDT1 in Huh7 cells.

(B) Dual luciferase assay results showing the suppressed transcription activity after mutation of the TFAP2A binding domains in Huh7 cells.

(C) Pie chart showing the expression peaks of deletion-up/amplification-down genes at different cell phases.

Students' t test, * $p < 0.05$, ** $p < 0.01$, *** $p < 0.001$. Means ± SEM from three independent experiments.

Suppl. Figure 4

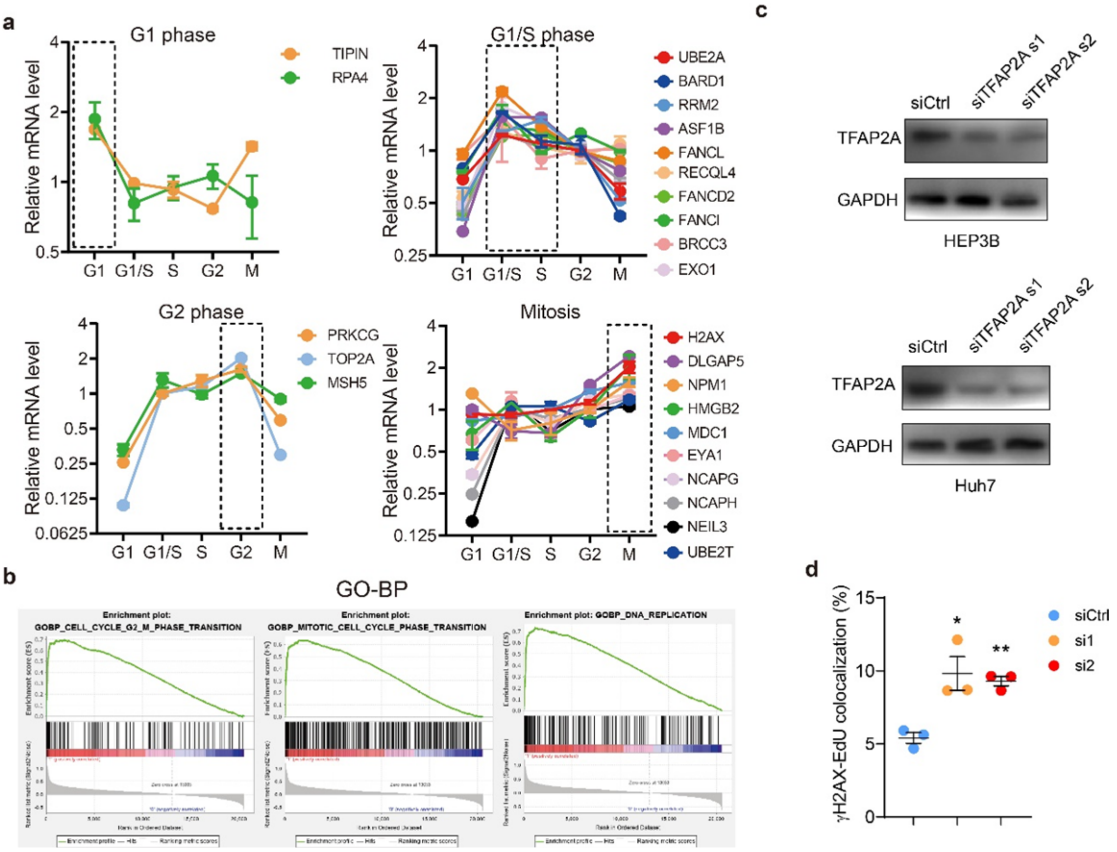

(A) Dot plot showing the enrichment of G2/M phases in patients expressing high levels of deletion-up genes.

(B) GSEA assay results showing the enrichment of G2/M phases in patients expressing high levels of deletion-up genes.

(C) Immunoblots showing the siTFAP2A efficacy in HEP3B and Huh7 cells.

(D) Quantification of immunofluorescence results in TFAP2A-depleted Huh7 cells. γH2AX-EdU colocalization was quantified with Cellprofiler.

Students' t test, * $p < 0.05$, ** $p < 0.01$, *** $p < 0.001$. Means ± SEM from three independent experiments.

Suppl. Figure 5

a

	ZINC ID	Affinity (kcal/mol)
Similar compounds N = 447 (top 10 in docking energy)	ZINC1911575176 (LEI110)	**-7.0**
	ZINC238873964	-6.0
	ZINC118703166	-5.6
	ZINC238873964	-5.5
	ZINC149968488	-5.3
	ZINC109990775	-5.3
	ZINC107800618	-5.0
	ZINC238017849	-5.0
	ZINC60415601	-4.8
	ZINC78991292	-4.3

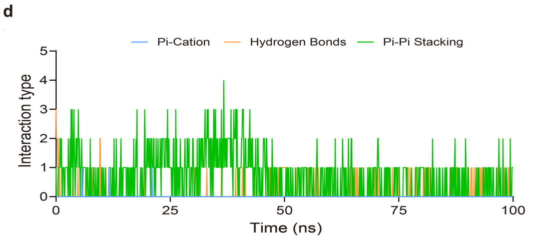

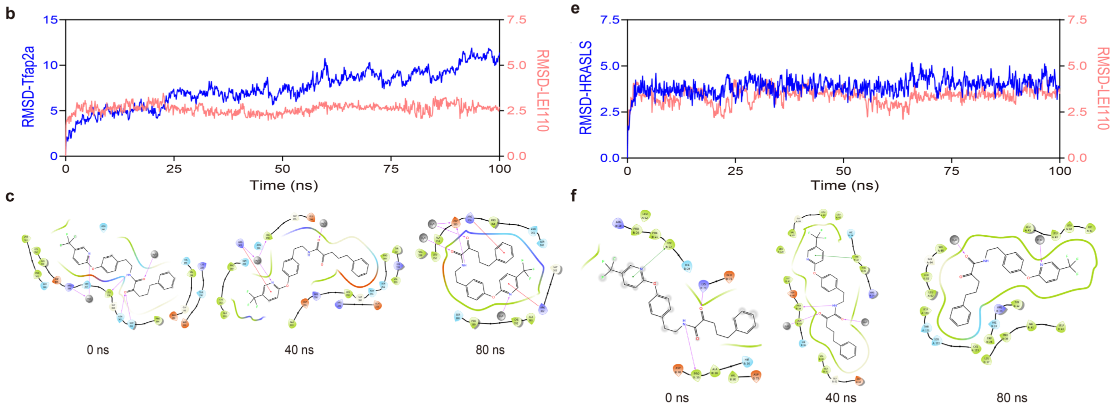

(A) Docking results of LEI110 and its analogs with TFAP2A in Autodock Vina.

(B-C) Molecular dynamic simulation results showing the stabilization of TFAP2A with LEI110.

(D) Quantification of interaction types between LEI110 and TFAP2A in the molecular dynamics simulations.

(E-F) Molecular dynamic simulation results showing very weak stabilization of HRASLS by LEI110.

Students' t test, * $p < 0.05$, ** $p < 0.01$, *** $p < 0.001$. Means ± SEM from three independent experiments.

Suppl. Figure 6

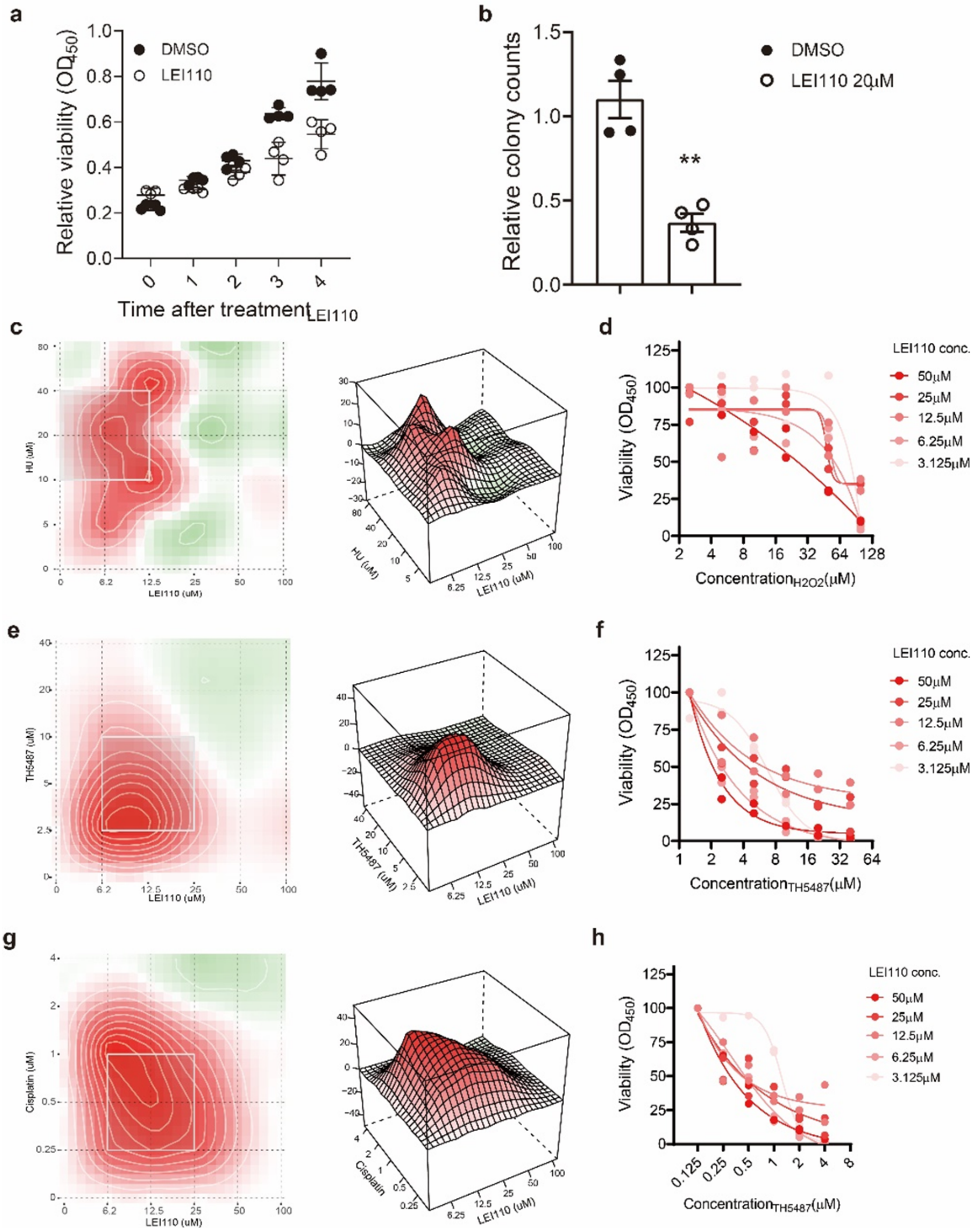

(A) Dot plot showing the suppressed proliferation of Huh7 cells by LEI110.

(B) Quantification of the clonogenic ability of Huh7 cells after LEI110 treatment (20μM).

(C-H) Synergy plot (C, E, G) and viability assay results (D, F, H) showing the sensitization of HEP3B cells towards different DNA damage-inducing reagents.

Students' t test, * $p < 0.05$, ** $p < 0.01$, *** $p < 0.001$. Means ± SEM from three independent experiments.

Suppl. Figure 7

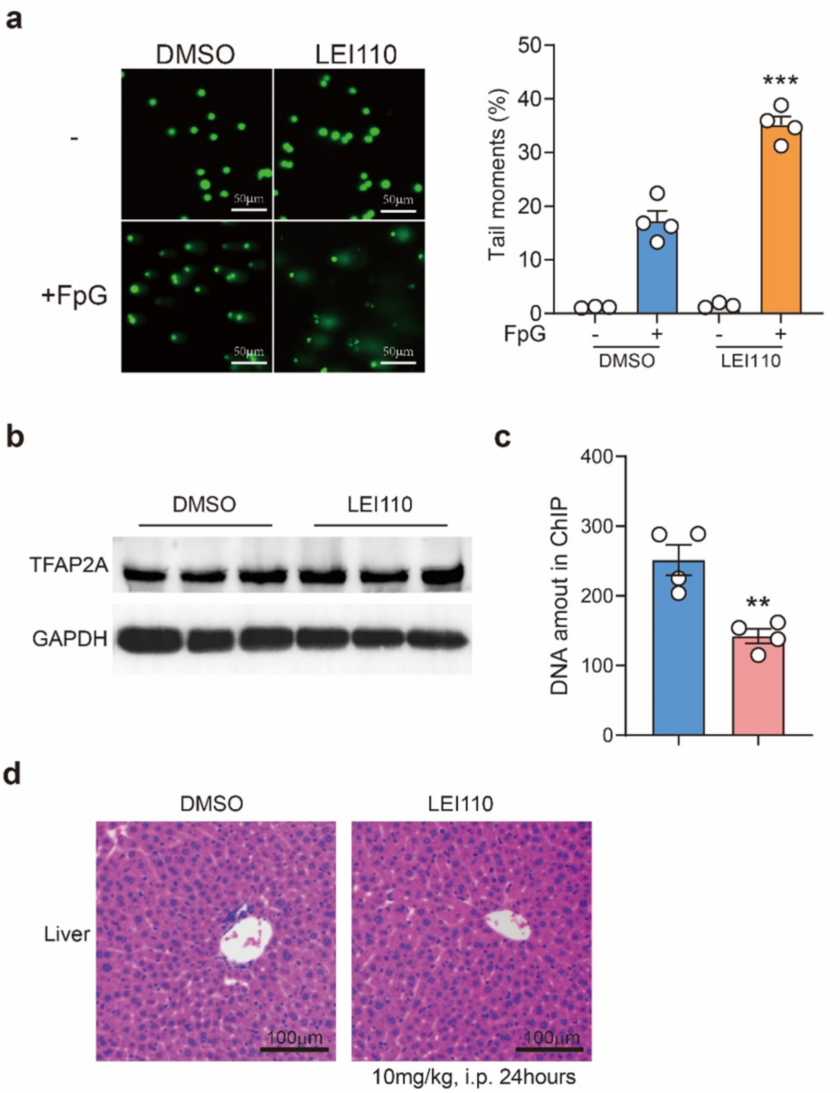

(A) Typical figures and quantifications of tail moments in the modified comet assay. HEP3B cells were treated with DMSO or LEI110 and the oxidized DNA lesions were recognized and excised by FpG.

(B) Immunoblot results showing the expression levels of TFAP2A after LEI110 or DMSO treatment. Cell lysates were harvested after 48 hours and antibodies targeting TFAP2A and GAPDH were used as internal control. And the experiments were performed in triplicates.

(C) Quantitation of DNA binding activity by TFAP2A after DMSO or LEI110 treatment. ChIP experiments were performed and the DNA concentrations were measured with nanodrop. The total amount of DNA was calculated with the total volume and concentrations of immunoprecipitated DNA.

(D) Typical figures for the Hematoxylin-eosin staining results in the livers of mice after LEI110 or DMSO treatment. Mice were injected with 10mg/kg LEI110, i.p., and liver tissues were harvested after 24 hours.

Students' t test, * $p < 0.05$, ** $p < 0.01$, *** $p < 0.001$. Means ± SEM from three independent experiments.

Suppl. Figure 8

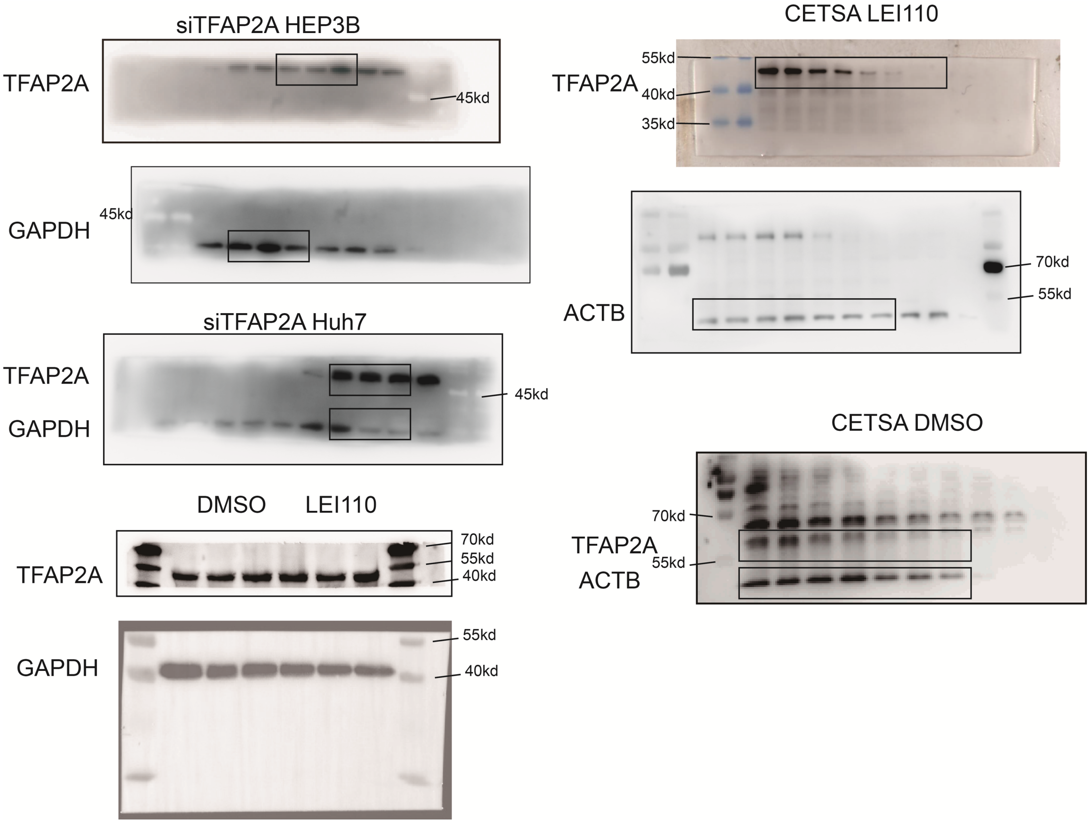

Suppl. Figure 8. Raw figures for the immunoblots in the study.
